# Smoldering myeloma presenting as progressive multifocal leukoencephalopathy: a case report

**DOI:** 10.1186/1752-1947-6-177

**Published:** 2012-07-02

**Authors:** Martina Troppmann, Roland Büttner, Michael Boewer, Bernd Salzberger

**Affiliations:** 1 Department of Internal Medicine I, University of Regensburg, Regensburg, Germany; 2 Department of Radiology, Hospital of Deggendorf/Mainkofen, Mainkofen, Germany

**Keywords:** Progressive multifocal leukoencephalopathy (PML), Polyomavirus JC (JCV), Cidofovir, Plasmacytoma

## Abstract

**Introduction:**

Progressive multifocal leukoencephalopathy is an opportunistic infection occurring in patients with severe cellular immunodeficiency. This case highlights the role of cellular immunodeficiency in the reactivation of John Cunningham virus in a case of an early stage plasmacytoma.

**Case presentation:**

A 76-year-old Caucasian woman presented with progressive left-sided hemiparesis, accompanied by hypoesthesia, hypoalgesia and neuropsychological symptoms. Magnetic resonance imaging demonstrated new hyperattenuating lesions in the right thalamus and left-sided subcortically. A polymerase chain reaction test revealed 4500 copies of John Cunningham virus-deoxyribonucleic acid/ml in cerebrospinal fluid. Human immunodeficiency virus infection was ruled out. A bone marrow biopsy showed an early stage immunoglobulin G-kappa plasmacytoma. Cidofovir (5mg/kg) weekly for three weeks was started. A significant improvement of her neuropsychological symptoms was achieved, but motor system and sensory symptoms did not change.

**Conclusions:**

This case shows a rapid course of progressive multifocal leukoencephalopathy with severe residual deficits. In the diagnostic workup of all patients with atypical neurologic symptoms or immunodeficiency, progressive multifocal leukoencephalopathy should be included as a differential diagnosis.

## Introduction

Progressive multifocal leukoencephalopathy (PML) is an opportunistic infection occurring in patients with severe cellular immunodeficiency. It is caused by reactivation of the John Cunningham (JC) polyomavirus. The prevalence of JC virus infection in the healthy population is estimated at 66% to 92% [1]. Primary infection usually does not result in clinical symptoms, but the virus persists in the kidney in a latent state. With immunodeficiency, however, the virus can reactivate, undergo genetic changes and lead to the clinically apparent neurologic manifestations of PML. Typically, PML is found in human immunodeficiency virus (HIV)-infected patients as an acquired immunodeficiency syndrome (AIDS)-defining illness, but organ and stem cell transplant patients, patients with hematological malignancies and patients treated with natalizumab are also at risk. Lytic infection of the myelin-producing glia cells leads to a multifocal demyelinization of cerebral white matter. The clinical manifestations depend on the localization of the lesions. Common symptoms are motor weakness, especially hemiparesis, visual deficits such as hemianopsia, and mental changes from circumscript single cognitive deficits up to dementia [2].

For the diagnosis of PML a magnetic resonance imaging (MRI) scan of the brain and cerebrospinal fluid examination are necessary. A cerebral MRI has a higher sensitivity in imaging of PML than cerebral computed tomography (CT) [3]. On T2-weighted images, MRI typically shows hyperattenuating lesions without mass effect [3]. The detection of JC virus (JCV) deoxyribonucleic acid (DNA) in cerebrospinal fluid by polymerase chain reaction (PCR) is needed for the diagnosis of PML. In some cases a biopsy may be necessary to either make a definite diagnosis or rule out other diseases, such as toxoplasmosis or lymphoma [4,5].

There is no standard therapy for PML. Improving the immunological status with highly active anti-retroviral therapy (HAART) is the essential therapeutic cornerstone in patients with HIV infection.

Therapy with alpha-interferon and cytarabine showed no clinical efficiency [6,7]. Cidofovir, a nucleotide analog of deoxycytidine monophosphate, shows *in vitro* activity against JCV but cidofovir has not been evaluated in a prospective randomized trial in patients with PML. Case series and reports do not demonstrate a clear benefit of therapy with cidofovir.

This case report demonstrates the occurrence of PML in a formerly healthy patient including treatment with cidofovir.

## Case presentation

**Figure 1:**
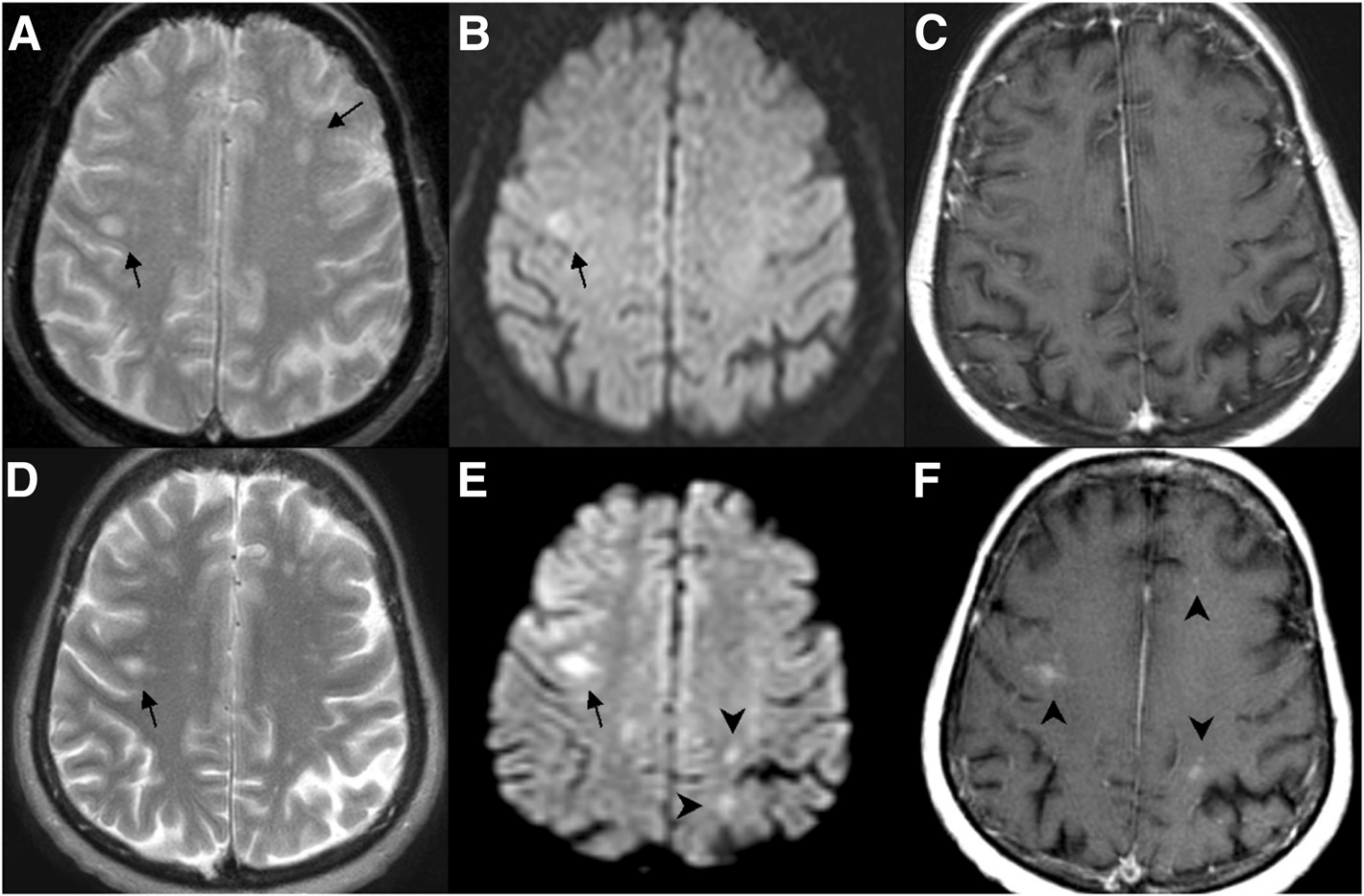
**In the initial scan, T2-weighted (A) axial magnetic resonance imaging scan demonstrates small foci of disease bilaterally (arrows).** The diffusion-weighted image (**B**) shows mild hyperintensity of the lesion on the right (arrow). The postgadolinium T1-weighted image (**C**) shows no significant enhancement. In the scan obtained three weeks later, the T2-weighted image (**D**) reveals a mild change in the lesion on the right (arrow). The diffusion-weighted image (**E**) reveals a marked higher signal intensity as in (**B**) (arrow) and shows new lesions left-sided subcortically (arrowheads). The postgadolinium T1-weighted image (**F**) now demonstrates marked progression of disease with patchy enhancement of lesions bilaterally (arrowheads).

A 76-year-old Caucasian woman from Bavaria, Germany was admitted to a primary care hospital with acute paresthesia of the left hand and face. Her clinical examination revealed a hemiparesis of the left part of the body, accompanied by hypoesthesia, hypoalgesia and neuropsychological symptoms. An initial cerebral CT showed no signs of acute ischemia or bleeding. Subsequent cerebral MRI revealed a subcortical lesion in the right hemisphere, first interpreted as lacunar infarction. Despite standard treatment for cerebral ischemia, the hemiparesis was progressive and she was transferred to a neurology unit. Control MRI scanning took place, and the T2-weighted analysis showed new hyperattenuating lesions in the right thalamus and left-sided subcortical regions. Other lesions located in both periventricular and subcortical white matter remained unchanged. Diffusion-weighted imaging now demonstrated abnormal diffusion, especially in the right thalamus. In many of those older and newer lesions, T1-weighted postgadolinium images revealed patchy and faint enhancement which was not found in the first MRI study (Figure 1). A cerebrospinal fluid examination excluded bacterial meningoencephalitis, Lyme disease, tick-borne encephalitis, Herpes-simplex-virus 1 and 2 and Creutzfeld-Jacob Disease. Due to the radiological appearance of the lesion, a PCR test for JCV-DNA was performed which revealed 4500 copies of JCV-DNA/ml in her cerebrospinal fluid (CSF) (Table1). Testing for HIV-infection was repeatedly negative.

**Table 1:** Cerebrospinal fluid examination

**parameter**	**value**	**reference value**
cell count	3.3/μl	0.0 to 0.4μl
protein	86mg/dl	15 to 50mg/dl
albumin	51.3mg/dl	13.9 to 24.6mg/dl
glucose	56mg/dl	–-
lactate	2.1mmol/L	1.1 to 2.1mmol/L
IgG	10.50mg/dl	0.48 to 5.86mg/dl
polyoma (JCV) DNA-PCR	4500 copies/ml	negative
protein 14-3-3	negative	negative
FSME (tick-borne encephalitis) – IgG and – IgM	negative	negative
Measles – IgG and – IgM	negative	negative
Lyme – IgG and – IgM	negative	negative

*Ig* immunoglobulin.

The differential blood cell count revealed severe relative lymphocytopenia with only 8% lymphocytes. Serum immunofixation demonstrated a monoclonal IgG type kappa with hypogammaglobulinemia. On bone marrow biopsy a diffuse plasma cell infiltration of 20% was found. She was then transferred to our hospital. Past medical history included bronchial asthma, arterial hypertension, cardiac arrhythmia and allergies to multiple drugs. Medication consisted of candesartan and hydrochlorothiazide 8/12.5mg once a day, clopidogrel 75mg once a day, verapamil 120mg once a day, and ipratropium bromide 250μg. She did not have fever, nausea, emesis or headache.

On physical examination she was awake and cooperative, oriented with regard to time, place, person and situation but had difficulty concentrating. Neurologic examination confirmed a left-sided hemiparesis, especially of the lower extremity*.*

Laboratory tests on admission are presented in Table2.

**Table 2:** Laboratory parameters on admission

**parameter**	**unit**	**value**	**reference value**
sodium	mmol/l	137	135 to 150
potassium	mmol/l	3.7	3.50 to 5.50
creatinine	mg/dl	0.82	0.50 to 0.90
CK	U/L	121	26 to 140
LDH	U/L	321 +	100 to 247
GOT	U/L	46 +	<35
GPT	U/L	37 +	<35
bilirubin	mg/dl	0.4	<1.00
protein	g/L	61.5 -	64 to 83
albumin	g/L	34.4 -	37.0 to 53.0
CRP	mg/L	46.22 +	<5.00
leucocytes	/nl	5.13	4.80 to 10.80
erythrocytes	/pl	4.73	3.80 to 6.10
hemoglobin	g/dl	15.1	11.7 to 15.7
thrombocytes	/nl	251	130 to 440

*CK* creatine kinase, *CRP C*-reactive protein, *GOT* serum glutamic oxaloacetic transaminase otherwise known as aspartate transaminase (AST) or aspartate aminotransferase (ASAT), *GPT* serum glutamic pyruvic transaminase otherwise known as alanine transaminase or alanine aminotransferase (ALAT), *LDH* lactate dehydrogenase.

Lymphocyte differentiation was done by fluorescence activated cell sorter (FACS) analysis. Results are shown in Table3.

**Table 3:** FACS-Analysis of lymphocyte subsets 29 April 2008

**parameter**	**unit**	**value**	**reference value**
leucocytes	/nl	4.75 -	4.80 to 10.80
lymphocytes	/nl	0.47 -	1.0 to 2.8
T- lymphocytes	% lymphos	27 -	55 to 83
B- lymphocytes	% lymphos	17	6 to 19
NK-cells	% lymphos	47 +	7 to 31
CD4+ cells	% lymphos	15 -	28 to 57
CD8+ cells	% lymphos	10	10 to 39
T- helper cells	/μl	72 -	300 to 1400
T- suppressor cells	/μl	48 -	200 to 900

*FACS* fluorescence activated cell sorter.

Further laboratory diagnostics showed a ß2-microglobulin of 2.92mg/l (0.7 to 1.8) and negative results for anti-HIV/HIV-antigen, hepatitis B virus surface (HBs)-antigen, anti-hepatitis B virus core (HBc) and anti-hepatitis C virus (HCV).

Skeletal involvement of plasmacytoma was ruled out by a radiographic series involving cervical, thoracic and lumbar spine, hips, femur, knees, upper arm and neighboring joints.

A diagnosis of PML secondary to immundeficiency was made. With the results of the bone marrow biopsy and serum immunofixation a diagnosis of an early stage plasmacytoma or smoldering myeloma was made. No other cause of immunodeficiency was found. Antineoplastic chemotherapy was not started immediately as lymphopenia might be enhanced and JCV-infection might be promoted at this time.

For PML we started cidofovir 5mg/kg intravenously weekly for three weeks. After the second administration of cidofovir a slight cognitive improvement was noticed. She started to be more active and communication improved. However, the hemiparesis did not regress. She was sent to a neurologic rehabilitation department two weeks after admission, where a third dose of cidofovir was given. The cognitive deficits recovered further. Short- and long-term memory, communication and orientation recovered fully. Hemiparesis of the left part of the body and incontinence for urine and stool persisted. She was not able to sit up, walk or eat on her own. No other manifestations of plasmacytoma developed during her follow-up. An antineoplastic chemotherapy was not initiated because the neurologic sequelae due to PML were considered not to be reversible and no other indication for chemotherapy was present. Nine months after diagnosis of PML she is in a stable, but severely impaired physical condition.

## Discussion

This patient not only had lymphocytopenia but more specifically a dramatic low CD4- (T-helper) cell count of 72/μl. In analogy to HIV-infection the risk of an opportunistic infection seems obvious. The occurrence of PML in patients with hematologic malignancies with low CD4-cell counts has been reported before. For instance, Re *et al*. reported a case of PML in a patient with hematological malignancy and a low CD4 cell count. One year after autologous bone marrow transplantation for therapy of a mantle cell lymphoma the patient developed neurological symptoms [8].

The differential diagnosis between cerebral ischemia and PML, as demonstrated in this case, can be difficult. Multiple neurologic complications and progressive disease should raise a suspicion and lead to further diagnostic procedures. MRI is the most sensitive imaging procedure in patients with PML. Definite diagnostic procedures are the detection of JC-DNA in cerebrospinal fluid or brain biopsy.

Our patient demonstrated a modest clinical response to antiviral therapy. A near complete recovery of neuropsychological symptoms could be achieved but her hemiparesis and the incontinence remained. Nine months after the diagnosis of PML she is still alive with stable neurological symptoms. Because of the clinical improvement follow up cerebrospinal fluid examinations and MRI scans were not performed.

Early stage plasmacytoma is the most likely cause of severe lymphocytopenia and immunodeficiency. Lymphocytopenia in this patient may have been further enhanced by the use of glucocorticoids for severe bronchial asthma just before the occurence of PML symptoms. An exact dose and timing of application could not be reconstructed.

## Conclusions

This case of a rapid progressive PML highlights the role of cellular immunodeficiency in the reactivation of JC-virus and the occurrence of PML. The clinical course may have been positively influenced by the use of cidofovir, although restitution of motor system functions could not be achieved. PML should be included as a differential diagnosis in all patients with cellular immunodeficiencies and rapid neurological deterioration. MRI scans may reveal typical or atypical findings and JC-virus can be detected by cerebrospinal fluid analysis or in brain biopsies.

## Consent

Written informed consent was obtained from the patient for publication of this case report and any accompanying images. A copy of the written consent is available for review by the Editor-in-Chief of this journal.

## Competing interests

The authors declare that they have no competing interests.

## Authors’ contributions

MT analyzed and interpreted the patient data regarding the infectious and hematological disease under the supervision of RB and BS. MB analyzed and interpreted the MRI images. BS helped to draft the final manuscript and was a major contributor in writing the manuscript. All authors read and approved the final manuscript.
